# Genetic polymorphisms, biomarkers and signaling pathways associated with septic shock: from diagnosis to therapeutic targets

**DOI:** 10.1093/burnst/tkae006

**Published:** 2024-05-06

**Authors:** Mingzheng Wu, Bobin Mi, Liu Liu, Haoli Ma, Cheng Jiang, Shan Jiang, Yulin Li, Yan Zhao

**Affiliations:** Emergency Medicine Center, Zhongnan Hospital of Wuhan University, 169 Donghu Road, Wuchang District, Wuhan 430061, China; Department of Orthopedics, Union Hospital, Tongji Medical College, Huazhong University of Science and Technology, 1277 Jiefang Avenue, Wuhan 430022, China; College of Education of Hubei University, 368 Youyi Avenue, Wuchang District, Wuhan 430062, Hubei Province, China; Department of Biological Repositories, Zhongnan Hospital of Wuhan University, 169 Donghu Road, Wuchang District, Wuhan 430071, Hubei Province, China; Emergency Medicine Center, Zhongnan Hospital of Wuhan University, 169 Donghu Road, Wuchang District, Wuhan 430061, China; Emergency Medicine Center, Zhongnan Hospital of Wuhan University, 169 Donghu Road, Wuchang District, Wuhan 430061, China; Emergency Medicine Center, Zhongnan Hospital of Wuhan University, 169 Donghu Road, Wuchang District, Wuhan 430061, China; Emergency Medicine Center, Zhongnan Hospital of Wuhan University, 169 Donghu Road, Wuchang District, Wuhan 430061, China

**Keywords:** Septic shock, Sepsis, Gene polymorphism, Pathogenic gene, Biomarker, Signaling pathway

## Abstract

Septic shock is a severe form of sepsis characterized by high global mortality rates and significant heritability. Clinicians have long been perplexed by the differential expression of genes, which poses challenges for early diagnosis and prompt treatment of septic shock. Genetic polymorphisms play crucial roles in determining susceptibility to, mortality from, and the prognosis of septic shock. Research indicates that pathogenic genes are known to cause septic shock through specific alleles, and protective genes have been shown to confer beneficial effects on affected individuals. Despite the existence of many biomarkers linked to septic shock, their clinical use remains limited. Therefore, further investigation is needed to identify specific biomarkers that can facilitate early prevention, diagnosis and risk stratification. Septic shock is closely associated with multiple signaling pathways, including the toll-like receptor 2/toll-like receptor 4, tumor necrosis factor-α, phosphatidylinositol 3-kinase/protein kinase B, mitogen-activated protein kinase, nuclear factor κB, Janus kinase/signal transducer and activator of transcription, mammalian target of rapamycin, NOD-like receptor thermal protein domain-associated protein 3 and hypoxia-induced-factor-1 pathways. Understanding the regulation of these signaling pathways may lead to the identification of therapeutic targets for the development of novel drugs to treat sepsis or septic shock. In conclusion, identifying differential gene expression during the development of septic shock allows physicians to stratify patients according to risk at an early stage. Furthermore, auxiliary examinations can assist physicians in identifying therapeutic targets within relevant signaling pathways, facilitating early diagnosis and treatment, reducing mortality and improving the prognosis of septic shock patients. Although there has been significant progress in studying the genetic polymorphisms, specific biomarkers and signaling pathways involved in septic shock, the journey toward their clinical application and widespread implementation still lies ahead.

Highlights

- Pathogenic genes are known to cause septic shock through specific alleles, while protective genes have been found to confer beneficial effects on affected individuals.
- Despite the existence of many biomarkers linked to septic shock, their clinical utilization remains limited.
- Septic shock is closely associated with multiple signaling pathways, including TLR2/TLR4, TNF-α, PI3K/AKT, MAPK, NF-κB, JAK/STAT, mTOR, NLRP3 and HIF-1.

## Background

Septic shock is a severe type of sepsis characterized by acute circulatory failure associated with infection. It is also a serious complication of burns and trauma. This condition affects millions of people worldwide each year and is related to high mortality, impaired quality of life for survivors and relatives, and high resource use [1]. The mortality rate of septic shock is ~38% in Europe and North America, and even higher rates have been reported in China [2,3]. Although mortality rates have decreased in recent years, the overall number of deaths continues to increase due to an increase in cases of sepsis [4]. The Sepsis-3 definition has been shown to be more effective at identifying septic individuals than the Sepsis-2 definition due to 92% overlap, fewer instances of septic shock and improved predictive validity [5]. Despite this, septic shock remains a significant complication that occurs in ~3.8% of patients with sepsis, highlighting the need for early diagnosis and timely intervention [6]. Early administration of antimicrobial therapy is crucial for septic shock treatment and should be given in adequate doses and be individualized for the shortest possible duration with the shortest possible rationalization [7]. Delayed administration of antibiotics has been shown to significantly affect mortality rates [8]. Septic shock patients who are treated with antibiotics within 3 h had a 35% increased risk of death for each hour of delayed antibiotic administration, emphasizing the importance of timely and effective intervention [9].

In this paper, we will review genetic polymorphisms, biomarkers and signaling pathways that could assist in the early and rapid identification of patients with septic shock and grading their shock status, and the advancement of preventive measures. This paper provides theoretical support for early diagnosis and precise treatment to ultimately improve the prognosis of septic shock and reduce morbidity and mortality.

## Review

### Understanding functional genomics

By comprehending the genomics of septic shock, we can improve prognostic risk factor assessment, expedite diagnosis and accurately predict an individual’s drug response. This approach can revolutionize medicine by significantly transforming disease prevention and treatment [10]. The genetic and genomic aspects of childhood septic shock are increasingly under investigation, and the accumulated data are expected to address ongoing clinical challenges and inform future strategies [11]. Studying the genomics of septic shock offers valuable insights into the structure, function and pathways associated with key genes and can aid in risk stratification [12]. Analysis of the GSE26440 and GSE33118 datasets yielded a total of 975 differentially expressed genes (DEGs), among which 30 were markedly upregulated. Six hub genes (CD177, CYSTM1, CLEC5A, MMP8, MCEMP1 and RGL4) exhibiting differential expression in septic shock patients were identified as diagnostic markers [13]. Recent bioinformatic analyses have successfully identified key genes associated with septic shock [12,14–29] (Table 1). These discoveries provide an opportunity to develop clinical tools for risk stratification and identify therapeutic targets in patients who survive septic shock.

**Table 1:** Key genes associated with septic shock

**Reference**	**Research objective**	**Dataset**	**Confirmed hub gene**
Grunwell *et al*. [14]	Differential expression of the Nrf2-linked genes in pediatric septic shock	GSE26440, GSE26378	PPARA, RXRA, NADPH- oxidase
Yang *et al*. [15]	Identification of key genes and pathways in septic shock children	GSE26440	GAPDH, TNF, EGF, MAPK3, IL-10, TLR4, MAPK14, IL-1b, PIK3CB, TLR2
Long and Yang [16]	Supporting vector machine classifier contributes to the diagnosis of pediatric septic shock	GSe26378, GSe26440, GSe13904, GSe4607	CYSTM1, S100A9, SLC2A14, STOM, UPP1, UTRN
Hu *et al*. [17]	Risk prediction in septic shock	GSE64457, GSE57065	WDR82, ASH1L, NCOA1, TPR, SF1, CREBBP
Mohammed *et al.* [12]	Identify the septic shock-related genes and functional groups	GSE66099	DDIT4, CCL3, PRG2, MT1M, CDC20, KIF20A, MAFF, EBI3, MELK, TOP2A, NUSAP1, RGL1, ARHGEF40, LOC254896, SLC46A2, TNFRSF10C
Wang *et al.* [18]	Transcriptomic markers in pediatric septic shock prognosis	GSE9692, GSE26378, GSE26440	OLFM4, MME, CXCR2, CEACAM8, ELANE
Zeng *et al*. [19]	Identify key biomarkers associated with sepsis and septic shock	GSE13904, GSE154918	MAPK14, FGR, RHOG, LAT, PRKACB, UBE2Q2, ITK, IL2RB, CD247
Yu *et al*. [20]	Study the pathogenesis of septic shock	GSE119217, GSE26378	CD63, C3AR1
Shi *et al.* [21]	Identification of transcriptomics biomarkers	GSE33118	MME, THBS1
Tang *et al*. [22]	Identify potential biomarkers and therapeutic targets of septic-shock- associated acute kidney injury	GSE30718, GSE57065	VMP1, SLPI, PTX3, TIMP1, OLFM4, LCN2, S100A9
Niu *et al.* [23]	Identify the key immune-related genes	GSE131411, GTEx	TLR8, PPP3CA, KRAS
Xu *et al*. [24]	Identification and verification of potential core genes in pediatric septic shock	GSE26440	MMP9, CEACAM8, ARG1, MCEMP1, LCN2, RETN, S100A12, GPR97, TRAT1
Jiang *et al.* [25]	Key genes for septic shock	GSE95233, GSE57065, GSE131761	FYN, CD247
Fan *et al*. [26]	Reveal potential diagnostic gene biomarkers of septic shock	GSE4607, GSE13904, GSE26378, GSE26440, GSE65682, GSE95233	KLRF1, UPP1, RAB13, KIF1B, CLEC5A, NARF, DUSP3, FCER1A, CACNA2D3, HMGN3, ECRP, HDAC4, LHFPL2, MGST1, ARHGEF18
Kong *et al*. [13]	Potential biomarkers in septic shock	GSE33118, GSE26440	CD177, CLEC5A, CYSTM1, MCEMP1, MMP8, RGL4
Zhao *et al*. [27]	Identify key biomarkers associated with sepsis and septic shock	GSE154918	ELANE, LCN2, IFI44
Wang *et al*. [28]	Explore potential biomarkers for septic shock	GSE57065, GSE95233	CD8A, CD247, CD3G, LCK, HLA-DRA
Yang *et al*. [29]	Validate the significant roles of CD3D and CD247 in septic shock	GSE33118, GSE142255	CD3D, CD247

### Influence of gene polymorphisms on prognosis

The exploration of genetic polymorphisms associated with susceptibility to septic shock has not only advanced clinical medicine but also opened new avenues in preventive medicine. A genetic polymorphism refers to the presence of two or more alleles at a particular locus within the same population, and the frequency of the disease-causing allele exceeds 0.01% [30]. In 1999, Kumar *et al*. [31] reported that the presence of additional genetic polymorphisms could contribute to the progression and final outcome of infections, sepsis syndromes and septic shock. Future identification and characterization of genetic polymorphisms linked to infection, sepsis syndrome and septic shock prognosis hold significant promise as prognostic tools. For instance, the interleukin (IL)-27-964A > G polymorphism enhances IL-27 expression, thereby promoting an inflammatory response induced by sepsis and ultimately leading to sepsis progression and poor prognosis [32]. These genes play various roles, and certain alleles act as pathogenic genes and contribute to septic shock, while others function as protective genes that exert beneficial effects on patients.

### Pathogenic genes and alleles

Septic shock mortality is highly heritable and is associated with pathogenic genes in non-surviving organisms (Table 2). Several pathogenic genes associated with septic shock are strongly implicated in the regulation of inflammatory factors and cytokines. These alleles play significant roles in the development and progression of septic shock. Previous studies have shown that the frequency of the tumor necrosis factor 2 (TNF2) allele with the TNF-α polymorphism was higher (12%) in patients with surgical infections in the surgical intensive care unit than in the general population. However, surgical intensive care unit patients with the TNF2 allele did not exhibit a higher incidence of septic shock or baseline TNF-α levels after infection. However, once infective shock occurred, patients carrying the TNF2 allele had higher mortality rates [33]. The TNF-α single nucleotide polymorphism (SNP) G/A includes the genotypes +489G/A, −308G/A, −238G/A and −376G/A [34]. Sepsis associated kidney injury is often related with an increased risk of mortality and morbidity. There were significant differences in the genotype frequency of TNF-α(-376 G/A) SNPS between S-acute kidney injury and non-acute kidney injury patients. Furthermore, the GA and AA genotypes are independent predictors of S-acute kidney injury [35]. The +252A/G polymorphism in the TNF-β gene alternates between the G (TNF-β-252G) allele and the A (TNF-β-252A) allele. Among these, the TNF-β-252A genotype (known as ‘TNF-β2’ in most studies) predicts a high risk of sepsis, and TNF-β-252AA is associated with the development of septic shock [36]. However, no significant differences in the toll-like receptor (TLR)4 Asp299Gly polymorphism were found in patients with sepsis [37]. Patients with the vacuolar protein sorting-associated protein 13D rs6685273 CC genotype exhibited an increase in 28-day mortality and increased organ failure compared to those with the CT/TT genotype. The vacuolar protein sorting-associated protein 13D rs6685273 C allele is associated with increased IL-6 production *in vitro* [38]. The prevalence of the IL-10-1082G allele is significantly higher in septic shock patients than in patients without shock. IL-10-1082G plays a crucial role in the susceptibility of patients with acute severe pancreatitis to septic shock [39]. Similarly, the IL20 rs2981573 G allele has been linked to an increased risk of death within 28 days and more organ dysfunction in patients with septic shock than the A allele [40]. In the context of septic shock, the −964AA genotype is more prevalent. Furthermore, the presence of the A allele is significantly related to increased mortality within 28 days in sepsis patients. Notably, individuals carrying the −964AA genotype exhibit significantly increased expression of IL-27 compared to carriers of the GA/GG genotype [32]. Genotyping of interleukin 1 receptor antagonist (IL1RN) polymorphisms has shown that the IL-1RN*2 genotype is associated with an increased risk of septic shock in children with acute lymphoblastic leukemia [41]. CD14 promoter gene polymorphisms have also been linked to an increased risk of septic shock, and the TT genotype is associated with significantly higher mortality [42].

**Table 2:** Pathogenic genes and alleles

**Reference**	**Pathogenic gene**	**Description**	**Pathogenic allele**
Tang *et al*. [33], Küçükaycan *et al.* [34], Al-Amodi *et al*. [35]	TNF-α	Tumor necrosis factor α	TNF2, TNF-α-376 GA and TNF-α-376 AA
Holmes *et al*. [36]	TNF-β	Tumor necrosis factor β	TNF-β-252A and TNF-β-252AA
Behairy *et al*. [37]	TLR	Toll-like receptor	TLR2 Arg753Gln
Nakada *et al*. [38]	VPS13D	Vacuolar protein sorting-associated protein 13D, which is associated with increased IL-6 production *in vitro*	rs6685273 C and rs6685273 CC
Zhang *et al*. [39]	IL-10	Interleukin-10	IL-10-1082G
Nakada *et al*. [40]	IL-20	Interleukin-20	rs2981573 GG
He *et al*. [32]	IL-27	Interleukin-27	IL-27-964AA
Zapata-Tarrés *et al*. [41]	IL1RN	Interleukin-1-receptor antagonist	IL-1RN*2
Gibot *et al*. [42]	CD14	Cluster of differentiation 14	TT
Madách *et al*. [43]	PAI-1	Plasminogen-activator inhibitor 1	4G/4G and 4G/5G
Cogulu *et al*. [44]	ACE	Angiotensin-converting enzyme	I/I
Nakada *et al*. [45]	AGTRAP	Angiotensin II type 1 receptor-associated protein	rs11121816 GG
Nakada *et al*. [46]	ADRB2	Beta(2)-adrenergic receptor	rs1042717 AA
Graebin *et al*. [47]	HLA-G	Human leucocyte antigen G	Exon 8 at the 3′ UTR
Toubiana *et al*. [48]	IRAK1	Interleukin receptor-associated kinase 1	IRAK1 variant haplotype
Peng *et al*. [49]	TREM-1	Trigger receptor-1	AA
Pérez-García *et al*. [50]	CEACAM7	Carcinoembryonic antigen-related cell adhesion molecule 7	rs1001578, rs10409040 and rs889365
O’Dwyer *et al*. [51]	DDAH II	Dimethylarginine dimethylaminohydrolase II	G at position −449 in the DDAH II gene
Rosier *et al*. [52]	CISH	Cytokine-inducible SRC homology 2 (SH2) domain protein	rs143356980
Li *et al*. [53]	lincRNA-NR_024015	Long non-coding RNA lincRNA- NR_024015	rs8506 TT
Nakada *et al*. [54]	SVEP1	Sushi, von Willebrand factor type A, EGF and pentraxin domain containing 1	c.2080A > C (p. Gln581His, rs10817033)
Sun *et al*. [55]	PECAM-1 Leu125Val	Platelet endothelial cell adhesion molecule-1 Leu125Val	CG and GG(rs668:C > G)
Paludo *et al*. [56]	SOD2	Superoxide dismutase 2	47C(rs4880)
Nakada *et al*. [57]	LNPEP	Leucyl/cystinyl aminopeptidase	rs4869317 TT
Vilander *et al*. [58]	SERPINA4	Serpin peptidase inhibitor, clade A, member 4	rs2093266
Vilander *et al*. [58]	SERPINA5	Serpin peptidase inhibitor, clade A, member 5	rs1955656
Cui *et al*. [59]	ADAM10	A disintegrin and metalloproteinase 10	rs653765 CC
Temple *et al*. [60]	HSPA1B	HSP70 gene	HSPA1B-179C > T
Clavier *et al*. [61]	PTPN1	Tyrosine-protein phosphatase non-receptor type 1	-
Clavier *et al*. [61]	ATF6	Activating transcription factor 6	-
Turrel-Davin *et al*. [62]	BID	BH3-interacting domain death agonist	-
Turrel-Davin *et al*. [62]	FAS	Fatty acid synthase	-
Hara *et al*. [63]	Mint3/Apba3	Munc18–1-interacting protein 3; human amyloid beta A4 precursor protein- binding family A member 3	-
Wang *et al*. [64]	NLRC4	NLR family CARD domain containing protein 4	-

Other pathogenic genes clearly associated with a higher risk of septic shock have specific alleles. The fibrinogen activator inhibitor 1 4G/5G and 4G/4G genotypes have been associated with a higher risk of septic shock and multiorgan dysfunction syndrome than the 5G/5G genotype [43]. In addition, insertion/deletion angiotensin-converting enzyme (ACE) gene polymorphisms have been linked to an increased risk of sepsis in I allele carriers [44]. The GG genotype of rs11121816, which is a negative regulator of the type 1 angiotensin II receptor, has been shown to be associated with a higher mortality rate in patients with septic shock [45]. Furthermore, the beta(2)-adrenergic receptor gene rs1042717 is a pure haplotype of CysGlyGln and is associated with increased mortality and more organ dysfunction in patients with septic shock [46]. Human leucocyte antigen G (HLA-G) gene polymorphisms have also been associated with the critical prognosis of patients, and sHLA-G5 levels predict survival in patients with septic shock [47]. In contrast, the interleukin receptor associated kinase 1 variant haplotype has been linked to the need for prolonged mechanical ventilation and a higher mortality rate in septic shock patients [48]. Plasma IL-6 concentrations were higher in patients with the triggering receptor expressed on myeloid cells-1 (TREM-1) gene polymorphism rs2234246 AA genotype than in patients with the GG genotype, and the carcinoembryonic antigen-related cell adhesion molecule 7 rs1001578, rs10409040 and rs889365 polymorphisms can influence mortality associated with septic shock [49,50]. The severity of organ failure, inflammation and early shock in severe sepsis have also been associated with increased levels of asymmetrical dimethyl arginine (ADMA), which may be influenced by the dimethylarginine dimethylaminohydrolase II gene polymorphism [51]. Disruptions in the cytokine-inducible SRC homology 2 domain protein pathway due to genetic variants may increase the risk of death in patients with sepsis [52], and the LincRNA-NR_024015 rs8506 TT genotype has been associated with an increased risk of sepsis [53].

Multiple SNPs are significantly correlated with an increased risk of mortality and organ dysfunction in patients with septic shock, including Sushi, von Willebrand factor type A, EGF and pentraxin domain containing 1 c.2080a > C (p.Gln581His) [54], the platelet endothelial cell adhesion molecule-1 gene Leu125Val [55], the 47C allele of the superoxide dismutase 2 gene (rs4880) [56], changes in genetic leucyl/cystinyl aminopeptidase pressinase [57], the SNP rs2093266 in serpin peptidase inhibitor, clade A, member 4 and the SNP rs1955656 in serpin peptidase inhibitor, clade A, member 5 [58]. Additionally, the a disintegrin and metalloproteinase 10 rs653765 polymorphism CC genotype may functionally affect a disintegrin and metalloproteinase 10 messenger RNA (mRNA) expression by upregulating its substrates [59].

Although the alleles of several pathogenic genes associated with septic shock have not been identified, the HSPA1B-179C > T polymorphism affects heat shock protein (HSP)70 production and is a crucial determinant of individual predisposition to a range of infectious and inflammatory diseases [60]. Gene expression variants of tyrosine-protein phosphatase non-receptor type 1 and activating transcription factor 6 have been partially associated with the development of septic organ failure and the expression of markers of endothelial dysfunction [61]. BH3-interacting domain death agonist and fatty acid synthase are upregulated in patients with septic shock [62], and deletion of the munc18–1-interacting protein 3 (Mint3)/amyloid beta precursor protein binding family A member 3 (Apba3) gene disrupts the function of macrophages in mice, increases resistance to lipopolysaccharide and prevents septic shock [63]. Silencing of the NLR family caspase recruitment domain-containing protein (CARD) domain containing protein 4 gene and its related pathways can inhibit inflammation and ameliorate lung injury in mice with septic shock [64].

### Protective genes and alleles

Although numerous genes have been correlated with an increased risk of mortality and organ failure in septic shock, certain alleles or associated genes have been shown to confer protection (Table 3). For example, the TNF-α + 489G/A SNP A allele may protect against sepsis-related mortality within 24 h after the onset of sepsis [65]. Other studies have identified polymorphisms in genes related to the TLR2 and TLR4 pathways downstream of the essential bridging protein myeloid differentiation factor 88 (MyD88) adapter-like [Ser180Leu, Toll-interleukin 1 receptor domain-containing adaptor protein (TIRAP) rs8177374], which appears to protect patients against many infectious agents. The TIRAP 180 L allele has been shown to increase the innate immune response to TLR4 and TLR2 ligands and is related to increased resistance to infection [66]. Other genes, such as ACE, have been shown to play important roles in host defense against invading pathogens. Analysis of insertion/deletion ACE gene polymorphisms by reverse hybridization analysis revealed that the DD genotype may have a positive effect on the development of sepsis in healthy children [44]. Similarly, the rs315952C functionally synonymous coding variant of the IL1RN gene is preferentially transcribed and expressed (IL1RA) and is significantly associated with improved survival, reduced mortality at 90 days post-adjustment and rapid recovery from shock [67]. Other genes, such as olfactomedin-4, are associated with an increased risk of organ failure and death. However, patients with the rs17552047 A allele and the rs1891944 TT genotype have been shown to have higher survival rates than patients with the rs1891944 CC/CT genotype and rs17552047 G allele [68]. Additionally, the Arg-304-His substitution, which is induced by the rs2230349 G-to-A mutation in G protein-coupled receptor kinase 5, has been shown to disrupt the function of G protein-coupled receptor kinase 5 and reduce the IκB-α/nuclear factor (NF)-κB-mediated inflammatory response, ultimately providing genetic protection against sepsis progression and susceptibility to mortality [69]. Finally, certain adrenocortical candidate genes are associated with differences in patient responses to hydrocortisone treatment. Specifically, patients with high glucocorticoid-induced transcript protein expression who are treated with hydrocortisone experienced faster shock remission than those in the placebo group, while patients with higher beta-hydroxysteroid dehydrogenase type 1 (BHSD1) expression who were treated with hydrocortisone achieved slower shock remission than those in the placebo group [70]. However, further research is needed to investigate the protective genes involved in septic shock. Understanding the mechanism and signaling pathway of these genes may facilitate the identification of therapeutic targets.

**Table 3:** Protective genes and alleles

**Reference**	**Protective gene**	**Description**	**Protective allele**
Georgescu *et al*. [65]	TNF-α	Tumor necrosis factor α	489G/A SNP A
Ferwerda *et al*. [66]	TLR	Toll-like receptor	TIRAP 180 L
Cogulu *et al*. [44]	ACE	Angiotensin-converting enzyme	DD
Meyer *et al*. [67]	IL1RN	Interleukin-1-receptor antagonist	rs315952C
Pérez-García *et al*. [68]	OLFM4	Olfactomedin 4	rs17552047 A and rs1891944 TT
Xu *et al*. [69]	GRK5	G Protein-coupled receptor kinase 5	rs2230349A
Cohen *et al*. [70]	GLCCI1	Glucocorticoid-induced transcript protein 1	-

### Clinical use of identifying differential biomarker genes

Identifying differential biomarker genes is vital for accurate diagnosis, stratification of risk and prognosis of septic shock. Advances in high-throughput sequencing techniques have significantly expanded the pool of genetic biomarkers under investigation. The association between sustained high procalcitonin (PCT) levels and intensive care unit mortality indicates that PCT clearance at 48 h could serve as a valuable prognostic biomarker. According to the Sepsis-3 definition, PCT is a reliable biomarker for predicting sepsis or septic shock [71]. The Pediatric Sepsis Biomarker Risk Model (PERSEVERE)-XP incorporates serum proteins selected from 148 mortality risk assessment genes and offers a combination of protein and mRNA biomarkers that can stratify mortality risk and contribute to clinical decision-making. Furthermore, PERSEVERE-XP significantly improved upon PERSEVERE, highlighting the involvement of tumor protein 53-related cellular division, repair and metabolism in the pathophysiology of septic shock [72].

A risk stratification tool is a reliable method for estimating mortality in adults with septic shock. A study selected five candidate biomarkers (IL-8, GZMB, HSPA1B, CCL3 and CCL4), along with initial lactate levels, age and chronic disease burden, as indicators [73]. Based on the definition of Sepsis-3, a combined biomarker approach that includes PCT, IL-6, pentraxin-3 and lactate showed promising results in predicting 28-day all-cause mortality in patients diagnosed with sepsis or septic shock. Furthermore, this approach outperformed the sequential organ failure assessment (SOFA) score in mortality prediction [71]. Currently, although there are a few biomarkers used in the clinic, septic shock still has high morbidity and mortality. Given the genetic susceptibility associated with septic shock, further research is needed to identify specific biomarkers that can aid in early prevention and diagnosis, which are crucial for effective management.

### Biomarkers of differential gene expression

The transcriptional changes associated with DEGs include inflammation, the defense response, cell motility and cytokine/chemokine-mediated signaling. As sepsis progresses to septic shock, many cytokines are recruited and participate in the inflammatory response (Table 4 and Table S1, see online supplementary material). Systemic sepsis results in the release of several cytokines, including TNF-α, which is a key cytokine that leads to septic shock. SNPs in the TNF gene promoter region result in differential TNF expression. Plasma TNF-α levels are significantly increased in patients with sepsis and septic shock [74]. The TNF-α and TNF-β genes are located adjacent to each other on chromosome 6 of the human leukocyte antigen class III cluster. In addition, other candidate genes associated with septic shock include IL1RN, IL-6, IL-10, CD-14, HSP, TLR-4 and TLR-2 [36]. We measured the mRNA expression of TNF-α, interferon γ (IFNγ), IL-4, IL-10 and IL-12p35, which are associated with the inflammatory response during infection, in three patient groups [75]. The results showed that the transcription of IL-12 p40 in the brains of the mice was significantly induced after intraperitoneal injection of lipopolysaccharide (LPS) and peaked 6 h after the injection. In contrast, constitutive expression of the il-12p35 gene in the brain is very low [76]. Serum concentrations of neutrophil gelatinase-associated lipid transport protein, IL-6, IL-8, IL-10 and resistin are high during severe sepsis [77]. IL-7 and IL-15 have been identified as prognostic biomarkers of sepsis and septic shock and are strongly correlated with inflammatory markers and mortality, particularly IL-7 [78]. Increased activation of the IL-23/IL-17 pathway has a detrimental effect on septicemia-induced lung inflammation. However, IL-12/IFN-γ activation of the IL-23/IL-17 pathway may reduce the severity of inflammatory events, and both pathways may serve as therapeutic targets for the treatment of septicemia-induced acute respiratory distress syndrome [79]. Administration of the IL-2 and JES6–1 mAb (IL-2/JES6) significantly increased the susceptibility of C57BL/6 mice to LPS-induced shock [80].

**Table 4:** Biomarkers associated with septic shock

**Biomarker**	**Description**	**Possible effects related to septic shock^a^**
TNF-α [33,74]	Tumor necrosis factor α	Early mediating anti-inflammatory response
TNF-β [36]	Tumor necrosis factor β	Early mediating many kinds of inflammation
IL-1 [41,118]	Interleukin 1	Attracting neutrophils, causing the release of inflammatory mediators
IL-2 [80]	Interleukin 2	Stimulating and maintain T lymphocyte differentiation and proliferation
IL-4 [75]	Interleukin 4	Stimulating the proliferation of activated B lymphocytes and T lymphocytes
IL-6 [71,77]	Interleukin 6	Stimulating the activation of B lymphocyte and T lymphocyte proliferation, stimulating liver cell synthesis of acute phase protein, participating in inflammatory response
IL-7 [78]	Interleukin 7	Anti-apoptotic and induces the proliferation of CD4+ and CD8+ T lymphocytes
IL-8 [71,77]	Interleukin 8	Attracting and activating neutrophils, releasing integrins (CD11b/CD18)
IL-10 [75,77]	Interleukin 10	Down-regulating inflammatory response, inhibiting the activation, migration and adhesion of inflammatory cells, and inhibiting the synthesis and releasing of inflammatory factors
IL-12 [75,76]	Interleukin 12	T lymphocytes and NK cells were induced to differentiate and proliferate to produce gamma-interferon
IL-15 [78]	Interleukin 15	Prompting the generation of mature NK cells in the bone marrow, playing an important role in the generation, cytotoxicity and survival of CD8+ T lymphocytes
IL-17 [79]	Interleukin 17	The expression of IL-6, IL-8 and ICAM-1 was induced
IL-23 [79]	Interleukin 23	Promoting CD4+ T lymphocyte proliferation and IL-17 and IFNγ production
CD14 [42]	Cluster of differentiation 14	Binding to the LPS/LBP complex and mediating the stimulating effect of LPS on cells
CD64 [81]	Cluster of differentiation 64	As a bridge connecting humoral immunity and cellular immunity, it has the functions of immune complex clearance, antigen presentation, inflammatory medium release, bacterial phagocytosis etc.
CD74 [82]	Cluster of differentiation 74	Presenting antigen and initiate immune response
CD127 [83]	Cluster of differentiation 127 is α chain of IL-7 receptor	Regulating the specific response of T lymphocytes to IL-7
CD177 [13]	Cluster of differentiation 177	Regulating the function and homeostasis of regulatory T cells
CD247 [25]	Cluster of differentiation 247	Inhibiting immune response and is associated with chronic inflammation
IFNγ [75]	Interferon γ	Disease-resistant protoorganisms activate macrophages
HSP [36]	Heat shock protein	Improving the stress ability of cells, especially the heat resistance ability
PCT [71]	Procalcitonin	It is not directly involved in the initiation of sepsis response, but can amplifying and aggravate the pathological process of sepsis
CRP [85]	C-reactive protein	Activating complement and strengthening phagocytosis plays an opsonate role
PTX3 [71,89]	Pentraxin 3	It is a pattern recognition receptor involved in the regulation of host immune responses
Lactate [71]	Marker of tissue hypoxia	Excessive aerobic glycolysis is stimulated by Na^+^K^+^ATPase during septic shock
Ang-2 [89]	Angiopoietin-2	Promoting angiogenesis and increases vascular permeability in ischemic and/or hypoxic environments
MCP1 [89]	Monocyte chemoattractant protein 1	Chemotactic monocytes
TREM-1 [90]	Triggering receptor expressed on myeloid cells-1	Triggering and amplifying the inflammatory response
IGHG1 [86]	Immunoglobulin heavy constant gamma 1	Enhancing the body’s immunity
NGAL [86]	Neutrophil gelatinase- associated lipocalin	Markers of acute renal function loss after septic shock
IL1R2 [86]	Interleukin 1 receptor II	Regulating inflammatory cytokines and chemokines
LTF [86]	Lactoferrin transfer protein	Anti-inflammatory reaction, has a strong antibacterial, antiviral effect
MMP8 [86]	Matrix metalloproteinase 8	A key enzyme that initiates the breakdown of ECM
OLFM4 [86]	Olfactomedin 4	OLFM4 negatively regulates the NF-κB pathway
TIMP2 [97]	Tissue inhibitor of metalloproteinase 2	Inhibiting metalloproteinases and protecting ECM
IGFBP-7 [97]	Insulin-like growth factor-binding protein 7	Regulating insulin-like growth factors, leading to glucose metabolism disorders and type 2 diabetes
HMGB1 [87]	High mobility group protein B1	Inducing a late inflammatory response
ROS [88]	Reactive oxygen species	Leads to oxidative stress and cell damage
HLA-DR [82]	Human leukocyte antigen-DR	Associated with antigen presentation to CD4+ helper T cells
CGRP [91]	Calcitonin gene-related peptide	Activation of adenylate cyclase increases intracellular cAMP and dilates blood vessels
NO [91,92]	Nitric oxide	Diastolic blood pressure and excess NO can damage cells and tissues
SAA [92]	Serum amyloid A	Activating complement and promoting phagocytosis
VEGF [93]	Vascular endothelial growth factor	Hypoxia of the tissue promotes the proliferation of blood vessels
sFLT [93]	Soluble Fms-like tyrosine	Binding to VEGF, anti-angiogenesis
MR-proADM [96]	Mid-regional proadrenomedullin	Dilating blood vessels and lowering blood pressure
MT [94]	Metallothionein	Free radical removal, heavy metal detoxification function
mtDNA [95]	Mitochondrial DNA	Carries a gene with a pathogenic mutation that stimulates an inflammatory response
Resistin [77]	Rich in cysteine, a peptide hormone derived from fat	Reducing the sensitivity of skeletal muscle cells, stem cells and fat cells to insulin
LCN2 [86]	Lipid carrier protein 2	Causing insulin resistance and various neurological diseases
GZMB [71]	Granuloenzyme B	First, the chain reaction of caspases is excited, causing target cell DNA degradation activities, and then cracking
CCL3 [71]	C-C motif ligand 3	Chemotaxis of monocytes, T cells, NK cells, dendritic cells, B cells and eosinophils were induced
CCL4 [71]	C-C motif ligand 4	Chemotaxis of T cells, monocytes and NK cells were induced

^a^References of possible effects related to septic shock can be found in the online supplementary material

Several cluster of differentiation (CD) molecules, such as CD14 and CD177, are closely linked to septic shock and play roles in inflammation [13,42]. Serum CD64 levels, proadrenomedullin levels and the SOFA score have been shown to be effective parameters for predicting the prognosis and mortality of septic shock. Because CD64 is more convenient and practical, it may be used as an alternative to the SOFA score [81]. According to the leukocyte gene profile, S100A8 and S100A12 gene expression decreased over time, whereas CD74 expression increased compared to that on D0. S100A8 plasma levels, gene expression and recovery were reduced. The change in CD74 gene expression was significantly correlated with the number of HLA-DR monocytes [82]. Increased levels of soluble plasma CD127 (sCD127, IL-7 receptor alpha chain) can identify patients with septic shock in subgroups at high risk of death. Validating this biomarker in larger patient cohorts would make it suitable for patient stratification in future clinical trials [83]. The results of a meta-analysis suggested that FYN could contribute to patient prognosis and that CD247 could be used to distinguish between patients with sepsis and patients with systemic inflammatory response syndrome (SIRS) [25]. However, the use of these CD molecules in clinical diagnosis and risk stratification requires further investigation.

Additionally, several biomarkers of differential gene expression have been associated with septic shock. Although the pathophysiological significance of the calcitonin gene has not been determined, it is widely used as a biomarker of sepsis worldwide. However, experimental results have shown that calcitonin gene-deficient mice are protected from septic shock and exhibit reduced pulmonary inflammation, suggesting a role in the pathogenesis of this disease [84]. C-Reactive protein levels were positively correlated with susceptibility to septic shock [85]. Six selected DEGs (IGHG1, IL1R2, LCN2, LTF, MMP8 and olfactomedin-4) can be used to distinguish infectious from non-septic shock [86]. High-mobility group box 1 is crucial for the development of shock-induced ALI [87]. The production of reactive oxygen species (ROS) by neutrophils plays a key role in organ dysfunction associated with sepsis and is widely recognized as the main cause of sepsis, which can progress to severe sepsis and septic shock [88]. Plasma levels of pentraxin-3, monocyte chemoattractant protein 1 and angiopoietin-2 are early biomarkers for assessing the severity of sepsis and septic shock [89]. Activation of the TREM-1 pathway is associated with septic shock outcomes, and data suggest that modulating this pathway in patients with activated TREM-1 might improve survival [90]. Plasma vasodilatory neuropeptide calcitonin gene-related peptide concentrations directly correlate with nitrite and nitrate levels at admission and at 2-h intervals in patients with septic shock. Calcitonin gene-related peptide synergistically interacts with NO as an essential mediator of hypotension in septic shock in humans [91]. Changes in serum amyloid A and NO levels have been linked to mortality in critically ill patients [92]. Vascular endothelial growth factor (VEGF) and its receptor soluble membrane-like tyrosine kinase (sFLT) are biomarkers of endothelial cell activation. In children with septic shock, increased levels of VEGF and sFLT have been observed in emergency departments; however, an increase in sFLT is related to poorer clinical outcomes, while an increase in VEGF is not significantly associated with prognosis [93]. Metallothionein is highly expressed in septic shock non-survivors who have lower serum zinc levels [94]. Increased plasma levels of mtDNA and inflammatory vesicle gene expression in monocytes are characteristic of patients with septic shock caused by multidrug-resistant bacteria [95]. Regardless of the degree of organ failure, the median regional adrenomedullin pro-peptide concentration has independent accuracy for predicting mortality, making it a promising candidate for the identification of early sepsis patients with moderate disease severity and a high risk of death [96]. Insulin-like growth factor binding protein 7 and tissue inhibitor of metalloproteinase-2 can be used to predict acute kindey injury, death or dialysis within 72 h [97]. Although several markers are used clinically, many specific markers need to be explored among key genes (Tables 1–3).

### Therapeutic targets of gene regulation and mechanisms

**Figure 1:**
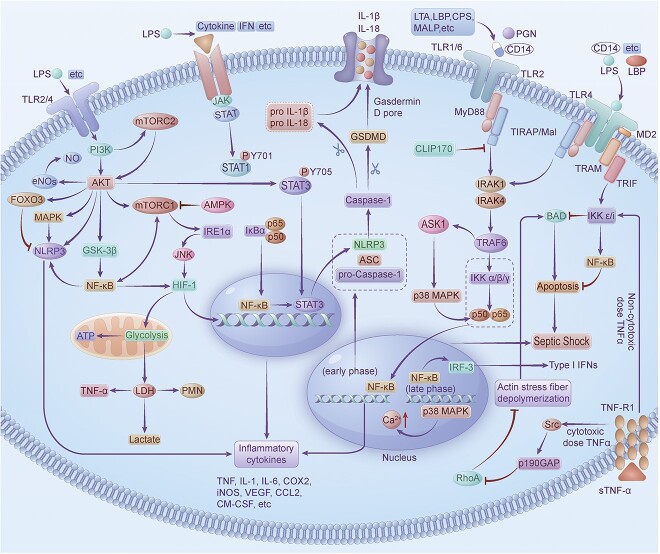
Main signaling pathways of septic shock. *TNF* Tumor necrosis factor, *TLR* toll-like receptors, *LTA* lipoteichoic acid, *CCL* C-C motif ligand, *HIF-1* hypoxy-induced-factor-1, *LPS* lipopolysaccharide, *PGN* peptidoglycan, *JNK* jun N-terminal kinase, *MALP-2* macrophage-activating lipopeptides 2 kDa, *LTA* lipoteichoic acid, *LBP* lipopolysaccharide-binding protein, *CPS* capsular polysaccharide, *iNOS* inducible nitric oxide synthase, *COX* cyclooxygenase-2, *eNOS* endothelial NO synthase, *NF-κB* nuclear transmutation of activated B cell, *ASK1* apoptosis signal-regulating kinase 1, *MAPK* mitogen-activated protein kinase, *PMN* polymorphonuclear neutrophil, IL interleukin, *JAK* Janus kinase, *STAT* signal transducer and activator of transcription, *PI3K* phosphatidylinositol 3-kinase, *AKT* protein kinase B, GSK-3β glycogen synthase kinase-3 beta, *FOXO* forkhead box O, *NLRP3* nucleotide-binding oligomerization domain-like receptor family pyrin domain-containing 3, *mTOR* mammalian target of rapamycin, *AMPK* adenosine monophosphate-activated protein kinase, *GSDMD* gasdermin D, *ASC* apoptosis speck-like protein, *MD2* myeloid differentiation factor 2, *MyD88* myeloid differentiation factor 88, *TIRAP* Toll-interleukin 1 receptor domain-containing adaptor protein, *TRAM* TRIF-related adaptor molecule, *TRIF* toll-interleukin-1 receptor domain-containing adaptor inducing interferon-β, *CLIP170* cytoplasmic linker protein 170, *IRAK* interleukin-1 receptor associated kinase, *TRAF6* tumor necrosis factor receptor associated factor 6, *BAD* bcl-2-associated death promoter, *IKK* IκB kinase complex, *IRF-3* IFN regulatory factor- 3, *IRE1α* inositol-requiring enzyme 1α, *LDH* lactic dehydrogenase, *VEGF* vascular endothelial growth factor, *CM-CSF* cellular macrophage-colony stimulating factor, *RhoA* ras homolog gene family A, *p190GAP* p190 GTPase accelerating protein, *IkBα* inhibitory kappa B alpha

Therapeutic targets for septic shock can be identified by studying genes and regulatory signaling pathways associated with this condition. We can examine biomarkers of differential gene expression. An analysis of health information showed that the first 10 hub genes in the protein-protein interaction (PPI) network of children with septic shock were the following upregulated DEGs: GAPDH, TNF, EGF, mitogen-activated protein kinase (MAPK3), MAPK14, IL-1β, IL-10, TLR2, TLR4 and PIK3CB. These genes are involved in one or more critical inflammatory pathways, such as the TNF, TLR, nuclear factor-κB (NF-κB), MAPK, phosphatidylinositol 3-kinase (PI3K)–protein kinase B (Akt), mammalian target of rapamycin (mTOR), Nod-like receptor and hypoxia-induced factor-1 (HIF-1) signaling pathways [15] (Figure 1). The mRNA expression levels of CD177, MMP8, CLEC5A, CYSTM1, MCEMP1 and RGL4 in peripheral blood mononuclear cells from patients with septic shock were higher than those in healthy donors. Enrichment analysis revealed that the ROS signaling pathway, hypoxia, the PI3K/AKT/mTOR signaling pathway, the NF-κB/TNF-α signaling pathway and the IL-6/Janus kinase (JAK)/signal transducer and activator of transcription 3 (STAT3) signaling pathway were significantly enriched. These findings suggest the involvement of these pathways in the pathophysiology of septic shock [13]. Therefore, understanding the regulation of these signaling pathways could provide therapeutic targets for the development of new drugs for treating sepsis or septic shock.

### Role of the TLR2/TLR4 signaling pathway in septic shock

During infection, TLR2 and TLR4, which are members of the TLR family, are widely recognized as the most important pattern recognition receptors because they can be activated by multiple antigens [37]. These membrane-bound proteins can effectively recognize invading organisms harboring pathogen-associated molecular patterns and damage-associated molecular patterns, trigger immune responses and protect the body from pathogenic infections [98]. TLR2 and TLR4 have been associated with the recognition of LPS, and TLR2 plays a central role in the recognition of gram-positive bacteria [99]. While TLR2 can recognize *Staphylococcus aureus* peptidoglycan (PGN), TLR4 can recognize gram-positive bacterial lipoprotein. The mycoplasmal lipopeptide macrophage-activating lipopeptides, 2 kDa and LPS are recognized by murine TLR2 and TLR4, respectively. The synergistic effects of TLR2- and TLR4-mediated signaling are involved in recognizing bacterial lipopeptides and lipoprotein compounds, which are structurally related to mycoplasmal lipopeptides [100]. The coreceptor CD14 facilitates TLR2/TLR4-mediated signaling in response to pattern recognition to mediate innate immunity, and Lgr4/Gpr48 negatively regulates CD14 expression [101] (Figure 1). TIRAP/MyD88 adapter-like is a convergent protein containing a Toll/IL-1 receptor (TIR) domain that interacts with TLR2 and TLR4 [102]. Cytoplasmic linker protein 170 interacts with the TLR2 and TLR4 junction protein TIRAP to induce the ubiquitination and subsequent degradation of TIRAP, thereby inhibiting LPS-induced IL-6 and TNF-α expression [103]. PGN, CD14 and TLR2 primarily recognize lipoteichoic acid, lipopolysaccharide-binding protein and capsular polysaccharide ligands of gram-positive bacteria, which stimulate downstream MyD88 to induce the release of proinflammatory cytokines (TNF-α, IL-1β, IL-6 and IL-12), chemokines (IL-8 and monocyte chemoattractant protein 1) and NO, and the expression of CD40, CD80 and CD86 [104–106]. TLR1 and TLR6 bind to different TLR2 adaptors; the MyD88 peer site is located at the D helix of TLR1, and the TIRAP recruitment site is located at TLR6 [107]. In addition to recognizing gram-negative LPS, TLR4 has complex activation mechanisms, and it recognizes HSPs released by host necrotic cells and requires the myeloid differentiation factor 2 protein as a coreceptor for activation [108]. TLR4 is the only TLR member that requires activation via two different intracellular signaling pathways: MyD88 and the TRIF (TIR domain containing adapter inducing interferon-β) adapter protein [109].

### Role of the TNF-α signaling pathway in septic shock

TNF-α plays an important role in inflammation during infections and autoimmune diseases. The TNF-α superfamily includes various members, such as a proliferation-inducing ligand, B-cell activating factor, and transmembrane activator and cyclophilin ligand interactor, which are critical regulators of B-cell functions. A proliferation-inducing ligand and B-cell activating factor are transmembrane proteins that are secreted by antigen-presenting cells and bind to three surface receptors expressed on B cells. Increased levels of the anti-inflammatory factor soluble transmembrane activator and cyclophilin ligand interactor receptor are associated with the severity of sepsis and poor prognosis, indicating a predominance of anti-inflammatory reactions and an unfavorable prognosis in septic patients [110]. Reducing the shedding of transmembrane TNF-α (tmTNF-α) may counteract the detrimental effects of secreted TNF-α (sTNF-α) and enhance the beneficial effects of tmTNF-α. This finding suggested that tmTNF-α and sTNF-α exerted opposite effects on septic shock [111]. The excessive secretion of TNF-α may trigger a systemic inflammatory response or septic shock, leading to life-threatening adverse effects. TNF-α is a ‘molecular trigger’ that is responsible for switching among the main TNF-α-dependent signaling pathways, such as inflammation, apoptosis and necroptosis [112,113]. Cytotoxic doses of TNF-α induce septic shock via the Src–p190 GTPase accelerating protein–ras homolog gene family A pathway [114] (Figure 1).

### Role of the NF-κB signaling pathway in septic shock

The transcription factor NF-κB represents a central downstream element of TLR-dependent signaling and has been implicated in regulating various biological phenomena and disease states, including the inflammatory response, apoptosis, cell proliferation, the stress response and innate immunity. Many NF-κB-dependent genes have been linked to the pathogenesis of sepsis and cardiac insufficiency in sepsis [115]. In mammals, the NF-κB transcription factor family consists of NF-κB1 (p105/p50), NF-κB2 (p/100/p52), p65 (RELA), RELB and REL, all of which are heterodimers, and the p65/p50 dimer is the most common [116]. NF-κB is a major regulator of immunity and inflammation, and it regulates the expression of many genes that are involved in inflammatory responses [117]. Two general types of NF-κB signaling pathways exist: the classical canonical pathway and the alternative non-canonical pathway. The classical (canonical) NF-κB pathway is triggered by TNF, IL-1 and Toll-like receptor ligands such as LPS or through conjugation with T-cell receptors and B-cell receptors [118] (Figure 1). Various signals activate NF-κB by degrading inhibitory kappa Bs, and activated NF-κB can bind to specific DNA sequences to initiate or regulate gene transcription, particularly immediate early genes that are involved in regulating the defense response [119].

### Role of the MAPK signaling pathway in septic shock

MAPK plays an important role in mediating activation and cytokine production during inflammation. The MAPK family includes evolutionarily conserved serine–threonine kinases that can be divided into the p38, c-Jun N-terminal kinase (JNK), extracellular signal-related kinase (ERK) and Big MAP kinase (BMK1) (also known as ERK5) subfamilies and represent the four classical MAPK pathways. The p38 and JNK pathways are mainly associated with stress responses, such as inflammation and apoptosis [120]. LPS induces intracellular ROS production and activates apoptosis signal-regulating kinase 1 (ASK1), a conserved mitogen-activated protein 3-kinase that plays a key role as an upstream regulator of MAPK. ROS-dependent activation of the TNF receptor-associated factor 6–ASK1–p38 pathway is selectively required for TLR4-mediated innate immunity [121]. ASK1 expression levels correlate with endothelial NO synthase (eNOS) expression in patients with septic shock, and interactions between MAPK and eNOS have been reported. Although LPS triggers the production of JNK-dependent cytokines requiring ASK1 activation, its effects on p38 permeability and activation are independent of ASK1 [122]. It has been reported that pharmacologically or genetically inhibiting p38 MAPK can lead to overactivation of the NOD-like receptor thermal protein domain-associated protein 3 (NLRP3) inflammasome, which triggers the activation of caspase 1 and increases in IL-1β and IL-18. Furthermore, a lack of p38 MAPK activity may cause an increase in soluble Ca^2+^ and excessive mitochondrial Ca^2+^ uptake, leading to excessive mitochondrial damage, which has been associated with NLRP3 inflammasome overactivation [123]. Proline isomerase peptidyl-prolyl isomerase NIMA-interacting-1 (Pin1) controls the expression of NLRP3, apoptosis speck-like protein (ASC) and caspase 1 during septic shock through phosphorylation of the p38 MAPK pathway. Pin1 deficiency inhibits the cleavage of gasdermin D and promotes macrophage death in response to LPS, which reduces the secretion of inflammatory cytokines, including IL-1β and IL-18 [124] (Figure 1). Therefore, p38 MAPK is able to promote apoptosis and autophagy, thereby improving the body’s ability to resist infection.

### Role of the JAK/STAT signaling pathway in septic shock

The JAK/STAT pathway is closely associated with sepsis and the resulting inflammatory response [125,126] (Figure 1). Sepsis-induced multiple organ failure is often accompanied by SIRS. Subsequently, compensatory anti-inflammatory response syndrome leads to sepsis-induced immunosuppression, late infection and an increased risk of mortality. The JAK/STAT-dependent signaling pathways are critical for both SIRS and compensatory anti-inflammatory response syndrome and are therefore crucial in the development of sepsis [127]. In response to exposure to LPS, the JAK/STAT pathway is activated, resulting in the phosphorylation of tyrosine residues at 705 and 701 on STAT3 and STAT1, respectively. Treatment with STAT3-specific inhibitors (e.g. static) blocks LPS-induced STAT3 tyrosine phosphorylation and inhibits LPS-induced production of IL-1β and IL-6 without affecting TNF-α production [128]. STAT3 is considered a key transcription factor in immune and inflammatory pathways. Continuous activation of NF-κB is maintained during chronic inflammation by prolonging the nuclear retention of RELA in transformed cells. STAT3 and NF-κB interact at multiple levels [129].

### Role of the PI3K/AKT signaling pathway in septic shock

The PI3K/AKT signaling pathway plays an important role in inflammation and infection. On the one hand, the PI3K/AKT signaling pathway can promote the occurrence and maintenance of the inflammatory response. On the other hand, the PI3K/AKT/HO-1 signaling pathway can protect against infection [130]. Sepsis-induced brain damage is associated with increased morbidity, mortality and cognitive impairment [131]. Multiple sepsis-related signaling pathways intersect with the PI3K/AKT pathway. For instance, serine-arginine protein kinase 1 (SRPK1) can inhibit sepsis-induced acute lung injury (ALI) by modulating the PI3K/AKT/ forkhead box O 3/NLRP3 pathway. SRPK1 overexpression can promote cell proliferation, inhibit apoptosis in primary human pulmonary microvascular endothelial cells, and alleviate sepsis-induced ALI *in vivo* through forkhead box O 3-mediated transcriptional inactivation of NLRP3 and inhibition of NLRP3 mRNA and protein expression. However, SRPK1 levels are decreased in patients with sepsis-induced ALI [132]. Exogenous administration of growth arrest-specific 6 can inhibit TNF-α release and apoptosis and attenuate the activation of NF-κB and MAPK through the Axl/PI3K/Akt pathway [133]. Glycolysis regulation is crucial for polymorphonuclear neutrophil (PMN) chemotaxis and phagocytosis during sepsis, and lactate dehydrogenase A is a key factor that downregulates PMN glycolysis. The PI3K/Akt–HIF-1α pathway can mediate the downregulation of lactate dehydrogenase A and affect the chemotactic and phagocytic functions of PMNs [134]. Resveratrol can protect the myocardium during sepsis by activating the PI3K/Akt/mTOR pathway and inhibiting the NF-κB signaling pathway and related inflammatory factors [135]. Notoginsenoside R1 is a promising compound that can protect the heart from septic shock by activating the estrogen receptor and PI3K/Akt pathways, inhibiting NF-κB, reducing the inflammatory state and alleviating apoptotic stress in the myocardium [136]. The adaptor proteins containing pleckstrin homology domain, phosphotyrosine domain and leucine zipper motif 1 (Appl1) and Appl2 are highly homologous and play critical roles in several signaling pathways. Appl2 is a key negative regulator of innate immune responses and forms complexes with Appl1 and PI3K to inhibit the PI3K/Akt/NF-κB signaling pathways [137]. Baicalin can regulate protein kinase R (PKR) by targeting ATP-binding and ATPase-active proteins in the PI3K/AKT/eNOS pathway, thereby exerting antiviral, anti-inflammatory, antitumor and antioxidant pharmacological effects [138]. Thymic stromal lymphopoietin can improve sepsis-induced hepatic injury by activating the PI3K/Akt/STAT3 pathway [139]. Heat shock proteins (Hsp70 and Hsp27) are regulated by positive feedback through the TLR4-PI3K/Akt-glycogen synthase kinase-3β pathway [140,141]. Cardiomyocyte apoptosis is a key factor that leads to myocardial dysfunction, and adiponectin can protect against this effect. Adiponectin can modify the Cx43/PI3K/Akt signaling pathway, preventing LPS-induced apoptosis during sepsis [142]. Administration of the TLR2 ligands PGN and Pam3CSK4 can alleviate cardiac dysfunction in septic mice through a TLR2/PI3K-dependent mechanism, and PGN administration leads to increased phosphorylation of Akt and glycogen synthase kinase-3β in the heart muscle [143] (Figure 1).

### Role of the mTOR signaling pathway in septic shock

The mTOR signaling pathway can regulate inflammatory responses in a variety of ways. mTOR is a serine/threonine kinase that forms the multiprotein complexes mTOR complex 1 and mTOR complex 2 by interacting with various proteins. Specific pharmacological inhibitors of the mTOR signaling pathway may be used for anti-inflammatory therapy in several inflammation-related diseases, such as cancer, neurodegenerative diseases, atherosclerosis, sepsis and rheumatoid arthritis [144]. Endoplasmic reticulum stress has been shown to be involved in the pathophysiology of many diseases by affecting apoptosis. The mTOR–Akt-inositol-requiring enzyme 1α-JNK signaling pathway mediates endoplasmic reticulum stress-induced CD4+ T-cell apoptosis in septic mice [145] (Figure 1). Esmolol, which is used to treat heart failure, alleviates myocardial injury induced by LPS by activating autophagy regulated by the adenosine monophosphate-activated protein kinase/mTOR/ULK1 signaling pathway [146]. ERK and PI3K phosphorylate mTOR, which controls cMaf translation in response to LPS-induced TLR4 signaling. Because cMaf can protect against septic shock [147], this information highlights the mTOR pathway as a potential therapeutic target for sepsis. Extracellular histones activate autophagy and apoptosis through the Sestrin2/adenosine monophosphate-activated protein kinase/ULK1-mTOR and AKT/mTOR pathways in human endodermal cells [148]. Inhibiting Na/K-ATPase is beneficial for activating Ca(^2+^)/CaMK/mTOR signaling [149]. However, further studies are needed to determine the specific roles and mechanisms of the mTOR signaling pathway in different inflammatory and infectious conditions.

### Role of the NLRP3 signaling pathway in septic shock

NLRP3 plays a pivotal role in the Nod-like receptor family. Activation of the NLRP3 inflammasome can trigger an inflammatory response by sensing pathogens or danger signals, resulting in the assembly of the NLRP3 complex and the activation of caspase-1. In response to a danger signal, NLRP3 interacts with and binds to the PYD domain of ASC. ASC recruits pro-caspase-1 via the same CARD domain to form the NLRP3 inflammasome. The activated NLRP3 inflammasome then cleaves pro-caspase-1 to form active caspase-1, which promotes the maturation of IL-1β and IL-18, ultimately leading to inflammation and cell death [150,151]. Interestingly, NLRP3 is an effective indicator of sepsis and septic shock and is no less accurate than the SOFA score. In fact, increased serum levels of NLRP3 (> 147.72 pg/ml) significantly increase the 30-day mortality rate of patients. Furthermore, NLRP3 has been shown to be helpful in predicting the risk of sepsis at an early stage, particularly in patients with septic shock. However, high levels of NLRP3 can also result in poor predictive outcomes for sepsis [152]. Studies have shown that a lack of p38 MAPK activity, which is associated with intracellular signal transduction, leads to an increase in soluble Ca^2+^ and excessive mitochondrial Ca^2+^ uptake, which leads to overactivation of the NLRP3 inflammasome. This overactivation, in turn, enhances caspase 1 activity, leading to increased production of IL-1β and IL-18 and additional mitochondrial damage [123]. The NLRP3 inflammasome plays a crucial role in detecting tissue damage and pathogen invasion through pattern recognition receptors, which are innate immune cell sensors. This process promotes the activation of the NF-κB and MAPK pathways, which ultimately leads to increased transcription of genes that encode NLRP3 inflammasome-associated proteins [151] (Figure 1). Therefore, the NLRP3 signaling pathway may be a key target for the treatment of inflammatory diseases.

### Role of the HIF-1 signaling pathway in septic shock

There is a relationship between the HIF-1 signaling pathway and inflammation. HIF-1 can activate downstream signaling pathways and participate in cell metabolism, proliferation and inflammatory responses under hypoxic conditions [153] (Figure 1). In the context of septic shock, hyperlactemia is often considered evidence of tissue hypoxia. Notably, inhibition of Na^+^K^+^ ATPase reduces muscle lactic acid levels [154]. Macrophages also play a critical role in the inflammatory response to sepsis. Mint3/APBA3 inhibits HIF-1 during normoxia in macrophages, thereby releasing FIH-1-mediated inhibition of HIF-1 activity. Mint3 regulates the FIH-1–HIF-1 pathway, which controls ATP production in macrophages [155]. The activation of HIF-1α induces not only the stabilization of inflammatory cells, including macrophages, neutrophils and dendritic cells, but also the production of cytokine storms in hypoxic and inflammatory states. This increase in cytokine and chemokine production leads to the accumulation of inflammatory cells at sites of inflammation and infection [156]. However, activated HIF-1 can also recruit inflammatory cells and induce specific cytokines (such as TNF, IL-10 and HIF1 derived from macrophages or IL-4 and IL-13 derived from mast cells, eosinophils or other cells), resulting in the generation of further inflammation, followed by the recurrence of hypoxia [157]. Additionally, the activation of HIF-1 and inducible nitric oxide synthase, as well as the upregulation of cyclooxygenase-2, are two important early responses that promote inflammation in the context of ischemia. These factors may cause organ damage through the rapid and excessive production of NO and prostaglandins [158].

In addition, recent research has shown that an imbalance in the gut flora plays a crucial role in sepsis and septic shock. Microbiome-targeted therapeutic strategies, such as epidemiogenesis, involves targeting inactivated microbial cells or cell components to exert antibacterial, immunomodulatory, antioxidant and antiproliferative effects, and can reduce the incidence of sepsis and improve the prognosis of patients with sepsis by regulating intestinal microbial metabolites, thereby improving intestinal barrier integrity and altering the composition of the intestinal microbiota. Epigenes may be superior to more traditional ‘organisms’, such as probiotics and prebiotics, and further basic research is needed to determine whether the prognosis of septic shock can be improved through these mechanisms [159]. In the context of sepsis, nuclear fragile X mental retardation-interacting protein 1-mediated nuclear autophagy is significantly activated through the PERK–activating transcription factor 4–CHOP pathway, thereby alleviating T lymphocyte apoptosis. Therefore, targeting nuclear fragile X mental retardation-interacting protein 1-mediated ribosomal phagocytosis may have important implications for reversing immunosuppression associated with sepsis complications [160].

## Conclusions

In summary, identifying differential gene expression during the development of septic shock can enable physicians to stratify the risk of patients in the early stages. Furthermore, conducting auxiliary examinations can help physicians identify therapeutic targets in relevant signaling pathways and lead to early diagnosis and treatment, reduced mortality and improved prognosis for patients with septic shock. Although many basic studies on the genetic polymorphisms, specific biomarkers and closely related signaling pathways involved in septic shock have been performed, much work is needed before clinical applications can be promoted.

## Supplementary Material

Table_S1_Supplementary_file_tkae006

## Abbreviations

ACE: Angiotensin-converting enzyme; AKT: Protein kinase B; ALI: Acute lung injury; Apba3: Human amyloid beta A4 precursor protein-binding family A member 3; ASC: Apoptosis speck-like protein; ASK1: Apoptosis signal-regulating kinase 1; CD: Cluster of differentiation; DEGs: Differentially expressed genes; eNOS: Endothelial NO synthase; ERK: Extracellular signal-related kinase; HIF-1: Hypoxia-induced-factor-1; HLA: Human leucocyte antigen; HSP: heat shock protein; IFNγ: Interferon γ; IL1RN: Interleukin 1 receptor antagonist; JAK: Janus kinase; JNK: Jun N-terminal kinase; LPS: Lipopolysaccharide; MAPK: Mitogen-activated protein kinase; NF-kB: nuclear factor-kB; Mint3: Munc18-1 interacting protein 3; mRNA: Messenger RNA; mTOR: Mammalian target of rapamycin; MyD88: Myeloid differentiation factor 88; NF-κB: nuclear transmutation of activated B cell; NLRP3: Nucleotide-binding oligomerization domain-like receptor family pyrin domain-containing 3; PCT: Procalcitonin; PGN: Peptidoglycan; PI3K: Phosphatidylinositol 3-kinase; PMN: Polymorphonuclear neutrophil; ROS: Reactive oxygen species; sFLT: Soluble membrane-like tyrosine kinase; SIRS: Systemic inflammatory response syndrome; SNP: Single nucleotide polymorphism; SRPK1: Serine-arginine protein kinase 1; STAT: Signal transducer and activator of transcription; NLRP3: NODlike receptor thermal protein domain associated protein 3; TIR: Toll/IL-1 receptor; TIRAP: Toll-interleukin 1 receptor domain-containing adaptor protein; TLR: toll-like receptor; TNF2: Tumor necrosis factor 2; TREM-1: Triggering receptor expressed on myeloid cells-1; TRIF: Toll-interleukin-1 receptor domain-containing adaptor inducing interferon-β; VEGF: Vascular endothelial growth factor; asymmetrical dimethyl arginine (ADMA); Bcl-2 homology 3(BH3); caspase recruitment domain-containing protein(CARD); NF-kappa-B inhibitor alpha(IκB-α); beta-hydroxysteroid dehydrogenase type 1 (BHSD1); sequential organ failure assessment (SOFA); monoclonal antibody(mAB); D0(day 0), mitochondrial DNA (mtDNA); Big MAP kinase(BMK1); protein-protein interaction (PPI); proline isomerase peptidyl-prolyl isomerase NIMA-interacting-1(Pin1); heme oxygenase1 (HO-1); anexelekto (Axl); protein kinase R (PKR); unc-51 like kinase 1(ULK1); factor inhibiting HIF-1 (FIH1); protein kinase R-like endoplasmic reticulum kinase(PERK); transcription factor C/EBP homologous protein (CHOP).
