# Supplementary material for: Genetic polymorphisms, biomarkers and signaling pathways associated with septic shock: from diagnosis to therapeutic targets
Source: Burns Trauma. 2024 May 6;12:tkae006. doi: 10.1093/burnst/tkae006 (PMC11074594; doi:10.1093/burnst/tkae006)
Supplement: Table_S1_Supplementary_file_tkae006 [file table_s1_supplementary_file_tkae006.doc]

**Table S1.** Biomarkers associated with septic shock

| **Biomarker** | **Description** | **Possible effects related to septic shock** |
| --- | --- | --- |
| TNF-α[34, 75] | Tumor necrosis factor α | Early mediating anti-inflammatory response [S1] |
| TNF-β[37] | Tumor necrosis factor β | Early mediating many kinds of inflammation [S2] |
| IL-1[42,121] | Interleukin 1 | Attracting neutrophils, causing the release of inflammatory mediators [S3] |
| IL-2[81] | Interleukin 2 | Stimulating and maintain T lymphocyte differentiation and proliferation [S4] |
| IL-4[76] | Interleukin 4 | Stimulating the proliferation of activated B lymphocytes and T lymphocytes [S5] |
| IL-6[72,78] | Interleukin 6 | Stimulating the activation of B lymphocyte and T lymphocyte proliferation, stimulating liver cell synthesis of acute phase protein, participating in inflammatory response [S5, S6] |
| IL-7[79] | Interleukin 7 | Anti-apoptotic and induces the proliferation of CD4+ and CD8+ T lymphocytes [S7] |
| IL-8[72, 78] | Interleukin 8 | Attracting and activating neutrophils, releasing integrins (CD11b/CD18) [S8] |
| IL-10[76, 78] | Interleukin 10 | Down-regulating inflammatory response, inhibiting the activation, migration and adhesion of inflammatory cells, and inhibiting the synthesis and releasing of inflammatory factors [S9] |
| IL-12 [76, 77] | Interleukin 12 | T lymphocytes and NK cells were induced to differentiate and proliferate to produce gamma - interferon [S10] |
| IL-15[79] | Interleukin 15 | Prompting the generation of mature NK cells in the bone marrow, playing an important role in the generation, cytotoxicity, and survival of CD8+ T lymphocytes [S11] |
| IL-17[80] | Interleukin 17 | The expression of IL-6, IL-8 and ICAM-1 was induced [S12] |
| IL-23[80] | Interleukin 23 | Promoting CD4+ T lymphocyte proliferation and IL-17, IFNγ production [S13] |
| CD14[43] | Cluster of differentiation 14 | binding to the LPS/LBP complex and mediating the stimulative effect of LPS on cells [S14] |
| CD64[82] | Cluster of differentiation 64 | As a bridge connecting humoral immunity and cellular immunity, it has the functions of immune complex clearance, antigen presentation, inflammatory medium release, bacterial phagocytosis and so on [S15] |
| CD74[83] | Cluster of differentiation 74 | Presenting antigen and initiate immune response [S16, S17] |
| CD127[84] | Cluster of differentiation 127 is α chain of IL-7 receptor | Regulating the specific response of T lymphocytes to IL-7  [S18] |
| CD177[13] | Cluster of differentiation 177 | Regulating the function and homeostasis of regulatory T cells [S19] |
| CD247[85] | Cluster of differentiation 247 | Inhibiting immune response and is associated with chronic inflammation [S20] |
| IFNγ[76] | Interferon γ | Disease-resistant protoorganisms activate macrophages [S21] |
| HSP[37] | Heat shock protein | Improving the stress ability of cells, especially the heat resistance ability [S22] |
| PCT[72] | Procalcitonin | It is not directly involved in the initiation of sepsis response, but can amplifying and aggravate the pathological process of sepsis [S23] |
| CRP[87] | C-reactive protein | Activating complement and strengthening phagocytosis plays an opsonate role [S24] |
| PTX3[72, 91] | Pentraxin 3 | It is a pattern recognition receptor involved in the regulation of host immune responses [S25] |
| Lactate[72] | Marker of tissue hypoxia | Excessive aerobic glycolysis is stimulated by Na+K+ ATPase during septic shock [S26] |
| Ang-2[91] | Angiopoietin-2 | Promoteing angiogenesis and increases vascular permeability in ischemic and/or hypoxic environments [S27] |
| MCP1[91] | Monocyte chemoattractant protein 1 | Chemotactic monocytes [S28] |
| TREM-1[92] | Triggering receptor expressed on myeloid cells-1 | Triggering and amplifying the inflammatory response [S29] |
| IGHG1 [88] | Immunoglobulin heavy constant gamma 1 | Enhancing the body's immunity [S30] |
| NGAL[88] | Neutrophil gelatinase- associated lipocalin | Markers of acute renal function loss after septic shock [S31] |
| IL1R2[88] | Interleukin 1 receptor Ⅱ | Regulating inflammatory cytokines and chemokines[S32] |
| LTF[88] | Lactoferrin transfer protein | Anti - inflammatory reaction, has a strong antibacterial, antiviral effect [S33] |
| MMP8[88] | Matrix metalloproteinase 8 | A key enzyme that initiates the breakdown of ECM [S34] |
| OLFM4[88] | Olfactomedin 4 | OLFM4 negatively regulates the NF-κB pathway [S35] |
| TIMP2[99] | Tissue inhibitor of metalloproteinase 2 | Inhibiting metalloproteinases and protect ECM [S36, S37] |
| IGFBP-7[99] | Insulin-like growth Factor-binding protein 7 | Regulating insulin-like growth factors, leading to glucose metabolism disorders and type 2 diabetes [S38] |
| HMGB1[89] | High mobility group protein B1 | Inducing a late inflammatory response [S39] |
| ROS[90] | Reactive oxygen species | It leads to oxidative stress and cell damage [S40] |
| HLA-DR[83] | Human leukocyte antigen-DR | Associated with antigen presentation to CD4+ helper T cells [S41] |
| CGRP[93] | Calcitonin gene-related peptide | Activation of adenylate cyclase increases intracellular cAMP and dilates blood vessels [S42] |
| NO[93, 94] | Nitric oxide | Diastolic blood pressure and excess NO can damage cells and tissues [S43] |
| SAA[94] | Serum amyloid A | Activating complement and promote phagocytosis [S44] |
| VEGF[95] | Vascular endothelial growth factor | The hypoxia of the tissue promotes the proliferation of blood vessels [S45] |
| sFLT[95] | Soluble Fms-like tyrosine | Binding to VEGF, anti-angiogenesis [S46] |
| MR-proADM [98] | mid-regional proadrenomedullin | Dilating blood vessels and lower blood pressure [S47] |
| MT [96] | Metallothionein | Free radical removal, heavy metal detoxification function [S48] |
| mtDNA[97] | Mitochondrial DNA | It carries a gene with a pathogenic mutation that stimulatings an inflammatory response [S49] |
| Resistin[78] | Rich in cysteine, a peptide hormone derived from fat | Reducing the sensitivity of skeletal muscle cells, stem cells and fat cells to insulin [S50] |
| LCN2[88] | Lipid carrier protein 2 | Causing insulin resistance, and various neurological diseases [S51] |
| GZMB[72] | Granuloenzyme B | First, the chain reaction of caspases is excited, causing the target cell DNA degradation activities, and then cracking [S52] |
| CCL3[72] | C-C motif ligand 3 | Chemotaxis of monocytes, T cells, NK cells, dendritic cells, B cells, and eosinophils were induced [S53, S54] |
| CCL4[72] | C-C motif ligand 4 | The chemotaxis of T cells, monocytes and NK cells were induced [S55, S56] |

**Supplementary references**

[S1] Park JY, Chung TW, Jeong YJ, Kwak CH, Ha SH, Kwon KM, et al. Ascofuranone inhibits lipopolysaccharide-induced inflammatory response via NF-kappaB and AP-1, p-ERK, TNF-α, IL-6 and IL-1β in RAW 264.7 macrophages. PLoS One 2017, 12:e0171322.

[S2] Watanabe M. Characteristics of TNF alpha- and TNF beta-induced fever in the rabbit. Jpn j physiol 1992, 42:101-116.

[S3] Broderick L, Hoffman HM. IL-1 and autoinflammatory disease: biology, pathogenesis and therapeutic targeting. Nat rev rheumatol 2022, 18:448-463.

[S4] Zhou X, Xing J, Tang X, Sheng X, Chi H, Zhan W. Interleukin-2 (IL-2) Interacts With IL-2 Receptor Beta (IL-2Rβ): Its Potential to Enhance the Proliferation of CD4+ T Lymphocytes in Flounder (Paralichthys olivaceus). Front Immunol 2020, 11:531785.

[S5] Habetswallner D, Pelosi E, Bulgarini D, Camagna A, Samoggia P, Montesoro E, et al. Activation and proliferation of normal resting human T lymphocytes in serum-free culture: role of IL-4 and IL-6. Immunology 2020, 65:357-364.

[S6] Tiggelman AM, Boers W, Linthorst C, Brand HS, Sala M, Chamuleau RA. Interleukin-6 production by human liver (myo)fibroblasts in culture. Evidence for a regulatory role of LPS, IL-1 beta and TNF alpha. J hepatol 1995, 23:295-306.

[S7] Vassena Lia,Miao Huiyi,Cimbro Raffaello,et al.Treatment with IL-7 prevents the decline of circulating CD4+ T cells during the acute phase of SIV infection in rhesus macaques.PLoS Pathogens.2012;8 (4)：e1002636.

[S8] Yoon BN, Choi NG, Lee HS, Cho KS, Roh HJ. Induction of interleukin-8 from nasal epithelial cells during bacterial infection: the role of IL-8 for neutrophil recruitment in chronic rhinosinusitis. Mediat inflamm 2010:813610.

[S9] Alexander AF, Kelsey I, Forbes H, Miller-Jensen K. Single-cell secretion analysis reveals a dual role for IL-10 in restraining and resolving the TLR4-induced inflammatory response. Cell Rep 2021, 36:109728.

[S10] Schmidt C, Brijs L, Cliquet P, De Baetselier P. Increased IL-12 P40 homodimer secretion by spleen cells during in vivo growth of the BW-19 T cell hybridoma accompanies suppression of natural immunity. Int j cancer 1998, 77:460-466.

[S11] Leśnik P, Janc J, Mierzchala-Pasierb M, Tański W, Wierciński J, Łysenko L (2023). Interleukin-7 and interleukin-15 as prognostic biomarkers in sepsis and septic shock: Correlation with inflammatory markers and mortality. Cytokine, 169:156277. (same with 79)

[S12] Kawaguchi M, Kokubu F, Kuga H, Matsukura S, Hoshino H, Ieki K, et al. Modulation of bronchial epithelial cells by IL-17. J allergy clin immun 2001, 108:804-809.

[S13] Basile JI, Kviatcovsky D, Romero MM, Balboa L, Monteserin J, Ritacco V, et al. Mycobacterium tuberculosis multi-drug-resistant strain M induces IL-17+ IFNγ- CD4+ T cell expansion through an IL-23 and TGF-β-dependent mechanism in patients with MDR-TB tuberculosis. Clin exp immunol 2017, 187:160-173.

[S14] Ryu JK, Kim SJ, Rah SH, Kang JI, Jung HE, Lee D, et al. Reconstruction of LPS Transfer Cascade Reveals Structural Determinants within LBP, CD14, and TLR4-MD2 for Efficient LPS Recognition and Transfer. Immunity 2017, 46:38-50.

[S15] Farias Mariela Granero,de Lucena Natália Pieruccini,Dal Bó Suzane,et al.Neutrophil CD64 expression as an important diagnostic marker of infection and sepsis in hospital patients.JOURNAL OF IMMUNOLOGICAL METHODS. 2014; 414：65-68.

[S16] Basha G, Omilusik K, Chavez-Steenbock A, Reinicke AT, Lack N, Choi KB, et al. A CD74-dependent MHC class I endolysosomal cross-presentation pathway. Nat immunol 2012, 13:237-245.

[S17] Sauler M, Zhang Y, Min JN, Leng L, Shan P, Roberts S, et al. Endothelial CD74 mediates macrophage migration inhibitory factor protection in hyperoxic lung injury. Faseb j 2015, 29:1940-1949.

[S18] Crawley Angela M,Angel Jonathan B.Expression of γ-chain cytokine receptors on CD8+ T cells in HIV infection with a focus on IL-7Rα (CD127).IMMUNOLOGY AND CELL BIOLOGY.2012;90 (4)：379-387.

[S19] Kim Myung-Chul,Borcherding Nicholas,Ahmed Kawther K,et al.CD177 modulates the function and homeostasis of tumor-infiltrating regulatory T cells.Nature communications.2021;12 (1)：5764.

[S20] Eldor R, Klieger Y, Sade-Feldman M, Vaknin I, Varfolomeev I, Fuchs C, et al. CD247, a novel T cell-derived diagnostic and prognostic biomarker for detecting disease progression and severity in patients with type 2 diabetes. Diabetes care 2015, 38:113-118.

[S21] Tsujimura H, Nagamura-Inoue T, Tamura T, Ozato K. IFN consensus sequence binding protein/IFN regulatory factor-8 guides bone marrow progenitor cells toward the macrophage lineage. J immunol 2002, 169:1261-1269.

[S22] Sharp FR, Massa SM, Swanson RA. Heat-shock protein protection. Trends neurosci 1999, 22:97-99.

[S23] Dahaba AA, Metzler H. Procalcitonin's role in the sepsis cascade. Is procalcitonin a sepsis marker or mediator? Minerva anestesiol 2009, 75:447-452.

[S24] Diaz Padilla N, Bleeker WK, Lubbers Y, Rigter GM, Van Mierlo GJ, Daha MR, et al. Rat C-reactive protein activates the autologous complement system. Immunology 2003, 109:564-571.

[S25] Jeon Jisu,Lee Yeji,Yu Hyeonseung,et al.Pseudomonas aeruginosa DnaK Stimulates the Production of Pentraxin 3 via TLR4-Dependent NF-κB and ERK Signaling Pathways.International journal of molecular sciences.2021;22 (9).

[S26] Levy Bruno,Gibot Sébastien,Franck Patricia,et al.Relation between muscle Na+K+ ATPase activity and raised lactate concentrations in septic shock: a prospective study.LANCET.2005;365 (9462)：871-875.

[S27] Ziegler Tilman,Horstkotte Jan,Schwab Claudia,et al.Angiopoietin 2 mediates microvascular and hemodynamic alterations in sepsis.JOURNAL OF CLINICAL INVESTIGATION.2013.

[S28] Kyriakides Themis R,Foster Matt J,Keeney Grant E,et al.The CC chemokine ligand, CCL2/MCP1, participates in macrophage fusion and foreign body giant cell formation.AMERICAN JOURNAL OF PATHOLOGY.2004;165 (6)：2157-2166.

[S29] Dubar Marie,Carrasco Kevin,Gibot Sebastien,et al.Effects of Porphyromonas gingivalis LPS and LR12 peptide on TREM-1 expression by monocytes.JOURNAL OF CLINICAL PERIODONTOLOGY.2018;45 (7)：799-805.

[S30] Scinicariello F, Engleman CN, Jayashankar L, McClure HM, Attanasio R. Rhesus macaque antibody molecules: sequences and heterogeneity of alpha and gamma constant regions. Immunology 2004, 111:66-74.

[S31] Cassidy H, Slyne J, Higgins M, Radford R, Conlon PJ, Watson AJ, et al. Neutrophil gelatinase-associated lipocalin (NGAL) is localised to the primary cilium in renal tubular epithelial cells - A novel source of urinary biomarkers of renal injury. Bba-mol basis dis 2019, 1865:165532.

[S32] Wan Y, Xiao H, Affolter J, Kim TW, Bulek K, Chaudhuri S, et al. Interleukin-1 receptor-associated kinase 2 is critical for lipopolysaccharide-mediated post-transcriptional control. J biol chem 2009, 284:10367-10375.

[S33] Zupin L, Polesello V, Segat L, Kamada AJ, Kuhn L, Crovella S. Association Between LTF Polymorphism and Risk of HIV-1 Transmission Among Zambian Seropositive Mothers. Curr hiv res 2018, 16:52-57.

[S34] Mazuchová J, Halašová E, Mazuch J, Šarlinová M, Valentová V, Franeková M, et al. Investigation of association between genetic polymorphisms of MMP2, MMP8, MMP9 and TIMP2 and development of varicose veins in the Slovak Population - pilot study. Physiol res 2020, 69:S443-s454.

[S35] Liu W, Yan M, Liu Y, Wang R, Li C, Deng C, et al. Olfactomedin 4 down-regulates innate immunity against Helicobacter pylori infection. P natl acad sci USA 2010, 107:11056-11061.

[S36] Fridman R, Bird RE, Hoyhtya M, Oelkuct M, Komarek D, Liang CM, et al. Expression of human recombinant 72 kDa gelatinase and tissue inhibitor of metalloproteinase-2 (TIMP-2): characterization of complex and free enzyme. Biochem j 1993, 289 ( Pt 2):411-416.

[S37] Costanzo L, Soto B, Meier R, Geraghty P. The Biology and Function of Tissue Inhibitor of Metalloproteinase 2 in the Lungs. Pulm med 2022:3632764.

[S38] Yan H, Li T, Wang Y, Li H, Xu J, Lu X. Insulin-like growth factor binding protein 7 accelerates hepatic steatosis and insulin resistance in non-alcoholic fatty liver disease. Clin exp pharmacol p 2019, 46:1101-1110.

[S39] Gentile LF, Moldawer LL. HMGB1 as a therapeutic target for sepsis: it's all in the timing! Expert opin ther tar 2014, 18:243-245.

[S40] Tur J, Pereira-Lopes S, Vico T, Marín EA, Muñoz JP, Hernández-Alvarez M, et al. Mitofusin 2 in Macrophages Links Mitochondrial ROS Production, Cytokine Release, Phagocytosis, Autophagy, and Bactericidal Activity. Cell Rep 2020, 32:108079.

[S41] Haug M, Schepp CP, Kalbacher H, Dannecker GE, Holzer U. 70-kDa heat shock proteins: specific interactions with HLA-DR molecules and their peptide fragments. Eur j immunol 2007, 37:1053-1063.

[S42] Herzog M, Scherer EQ, Albrecht B, Rorabaugh B, Scofield MA, Wangemann P. CGRP receptors in the gerbil spiral modiolar artery mediate a sustained vasodilation via a transient cAMP-mediated Ca2+-decrease. J membrane biol 2002, 189:225-236.

[S43] Moroz LL, Norby SW, Cruz L, Sweedler JV, Gillette R, Clarkson RB. Non-enzymatic production of nitric oxide (NO) from NO synthase inhibitors. Biochem bioph res co 1998, 253:571-576.

[S44] Vollmer Almut H,Gebre Makda S,Barnard Dale L.Serum amyloid A (SAA) is an early biomarker of influenza virus disease in BALB/c, C57BL/2, Swiss-Webster, and DBA.2 mice.ANTIVIRAL RESEARCH.2016;133：196-207.

[S45] Du Peizhao,Dai Fangjie,Chang Yaowei,et al.Role of miR-199b-5p in regulating angiogenesis in mouse myocardial microvascular endothelial cells through HSF1/VEGF pathway.ENVIRONMENTAL TOXICOLOGY AND PHARMACOLOGY.2016;47：142-148.

[S46] Altiok Eda I,Santiago-Ortiz Jorge L,Svedlund Felicia L,et al.Multivalent hyaluronic acid bioconjugates improve sFlt-1 activity in vitro. BIOMATERIALS. 2016;93：95-105.

[S47] Figueira L, Israel A. Role of cerebellar adrenomedullin in blood pressure regulation. Neuropeptides 2015, 54:59-66.

[S48] Sato M, Bremner I. Oxygen free radicals and metallothionein. Free radical bio med 1993, 14:325-337.

[S49] Zhang Q, Wei J, Liu Z, Huang X, Sun M, Lai W, et al. STING signaling sensing of DRP1-dependent mtDNA release in kupffer cells contributes to lipopolysaccharide-induced liver injury in mice. Redox Biol 2022, 54:102367.

[S50] Rawal K, Purohit KM, Patel TP, Karont N, Gupta S. Resistin mitigates stemness and metabolic profile of human adipose-derived mesenchymal stem cells via insulin resistance. Cytokine 2021, 138:155374.

[S51] Wu D, Wang X, Han Y, Wang Y. The effect of lipocalin-2 (LCN2) on apoptosis: a proteomics analysis study in an LCN2 deficient mouse model. BMC Genomics 2021, 22:892.

[S52] Pardo J, Gálvez EM, Koskinen A, Simon MM, Lobigs M, Regner M, et al. Caspase-dependent inhibition of mousepox replication by gzmB. PLoS One 2009, 4:e7512.

[S53] Schaller TH, Batich KA, Suryadevara CM, Desai R, Sampson JH. Chemokines as adjuvants for immunotherapy: implications for immune activation with CCL3. Expert rev clin immu 2017, 13:1049-1060.

[S54] Uyangaa E, Kim JH, Patil AM, Choi JY, Kim SB, Eo SK. Distinct Upstream Role of Type I IFN Signaling in Hematopoietic Stem Cell-Derived and Epithelial Resident Cells for Concerted Recruitment of Ly-6Chi Monocytes and NK Cells via CCL2-CCL3 Cascade. Plos pathog 2015, 11:e1005256.

[S55] Ikeno Y, Ohara D, Takeuchi Y, Watanabe H, Kondoh G, Taura K, et al. Foxp3+ Regulatory T Cells Inhibit CCl4-Induced Liver Inflammation and Fibrosis by Regulating Tissue Cellular Immunity. Front Immunol 2020, 11:584048.

[S56] Kumari S, Shivam P, Kumar S, Jamal F, Singh MK, Bimal S, et al. Leishmania donovani mediated higher expression of CCL4 induces differential accumulation of CD4+CD56+NKT and CD8+CD56+NKT cells at infection site. Cytokine 2018, 110:306-315.
